# Medical Kitchen: Transdisciplinary Clinical Skills Training

**DOI:** 10.1111/tct.70065

**Published:** 2025-03-08

**Authors:** Jakub L. Radzikowski, Natasha Houghton, C. Sofia Chacon, Oliver Armstrong‐Scott, Jozef Youssef, Alan C. Spivey, Aynkaran Dharmarajah, Roger Kneebone

**Affiliations:** ^1^ Department of Chemistry, Faculty of Natural Sciences Imperial College London London UK; ^2^ Department of Surgery and Cancer, Faculty of Medicine Imperial College London London UK; ^3^ Kitchen Theory Ltd London UK; ^4^ School of Medicine, Faculty of Medicine Imperial College London London UK

**Keywords:** clinical skills training, medical education, transdisciplinary simulation

## Abstract

**Background:**

Medical Kitchen is an innovative transdisciplinary simulation aimed at helping 2nd‐year medical students at Imperial College London transition from declarative to procedural knowledge acquisition and prepare them for learning clinical skills, grounded in established psychomotor skills development theories, including Kovacs' definitions and the Fitts and Posner's model.

**Approach:**

It employs a transdisciplinary simulation approach that blends professional gastronomy with medical training. Designed initially in response to the 2020 Covid‐19 pandemic, the course offers a scalable and replicable model that includes mechanisms for peer feedback and reflective exercises.

**Evaluation:**

The responses for these exercises were analysed thematically to gauge the course's effectiveness. Findings suggest that the Medical Kitchen succeeds in creating a low‐stakes, safe learning environment that not only enhances practical skill learning but also emphasises the crucial non‐technical skills needed in medical practice.

**Implications:**

The Medical Kitchen represents a forward‐thinking strategy for medical educators seeking to improve procedural skill training, warranting further research into the long‐term impacts of the course on student outcomes.

## 1 Background

Medical students must master basic clinical procedures during their undergraduate studies. As it is ethically impermissible for students to practice such skills on patients until they have reached a sufficient level of competence, this practice requires a safe environment like clinical skills labs [1]. Such a shift from ward‐based learning to central faculty teaching increases the burden on faculty resources [2], limiting the amount of learning opportunities. Moreover, due to the structure of the curriculum, procedural knowledge (‘knowing‐*how*’) receives less attention than declarative knowledge (‘knowing‐*that*’) during early undergraduate learning. Consequently, the students may feel unprepared when beginning clinical skills training.

The acquisition of procedural knowledge is a complex process. We adopted Kovacs definition of psychomotor skills as ‘mental and motor activities required to execute a manual task’ [3]. Repetition is crucial for developing psychomotor skills [4, 5]. Expertise development requires repeated engagement in structured practice, preferably under the guidance of an expert, and captured by Ericsson's term ‘sustained deliberate practice’ [5]. Reflection, whether before, during or after a task, is essential in clinical skills education [6]. Peer feedback is valuable, especially when it is relevant and well‐informed [2, 7, 8].

These processes are included in the major models of psychomotor development, of which Fitts' and Posner's are widely used in health education [9], for example, for surgeons [10]. They frame psychomotor skills acquisition as a phased process.

1. Cognitive phase: Students learn the basics, often making mistakes and showing inconsistent performance. They require repetition, demonstrations and instructor feedback.
2. Associative phase: As learners become more consistent and make fewer mistakes, they begin to self‐correct and focus for longer periods.
3. Autonomous phase: Students can perform tasks effortlessly and consistently, allowing them to manage complex situations, communicate effectively and address complications in clinical settings.

## 2 Approach

We utilised these theories to support 2nd‐year medical students during the transition between declarative knowledge and procedural knowledge development and familiarise them with the process of learning procedural knowledge to design a transdisciplinary practical skills introductory course, through which students develop a transferable approach to deliberate practice and learning new psychomotor skills. The course initially launched remotely due to the COVID‐19 pandemic. It later transitioned to a hybrid format and is now fully in‐person. The intended learning outcomes of the course can be found in Table 1, and the course manual is available in the Supporting Information.

**TABLE 1:** Intended learning outcomes.

ILO no.	ILO
1	Interpret and follow protocols for an unfamiliar procedure with an appropriate level of planning and forward thinking before beginning practical work
2	Handle equipment—safely prepare, use and clean potentially hazardous equipment
3	Demonstrate safe and hygienic working practices using aseptic technique where appropriate
4	Practice meticulous technique with a high level of dexterity, working carefully to manipulate tools consistently
5	Communicate appropriately while completing the procedure describing it in real time
6	Document outcomes accurately, in appropriate details and in an organised manner
7	Reflect on constructive feedback from self‐reflection, peers and tutors—learn from errors, seeing them as part of the process of skills development

Transdisciplinary learning integrates seemingly unrelated disciplines. The ability to learn psychomotor skills differs from performing a specific skill [11]. Removing the demand for factual knowledge in the home discipline may decrease intrinsic cognitive load by shifting attention from technical success to aspects like patient perceptions and improving skills such as preparation, communication and empathy.

Transdisciplinary simulation can provide a safe learning environment [12]. Learning unrelated procedures is ‘low stakes’, allowing students to fail, question and reflect without harming self‐efficacy. We selected professional gastronomy as the parallel discipline. Chefs share common ground with clinicians in communication, preparation (*mise‐en‐place)* [13], hygiene, precision, dexterity, etc. Both domains require personal protective equipment and specialist tools that are potentially dangerous and demanding to master. Parallels are presented in Table 2. Further advantages include the opportunity for lone students to practise remotely, as most dwellings are equipped for cooking. Gastronomy has previously supported transitions from high school to university chemistry [14].

Transdisciplinary simulation can provide a safe learning environment. Learning unrelated procedures is ‘low stakes’, allowing students to fail, question and reflect without harming self‐efficacy.

**TABLE 2:** Similarities between medicine (performing a clinical procedure) and gastronomy (turning vegetables).

Aspect	Medicine—clinical procedures	Gastronomy
Main hazards	Contamination or infection	Cross‐contamination of foods
Personal hygiene	Washing hands according to an established procedure	Washing hands according to an established procedure
Personal protective equipment	Gloves, or sterile gloves, mask and gown as required	Apron, protective jacket, protective shoes, gloves (non‐sterile)
Preparing the workplace	Establishing a sterile field, setting out all the necessary equipment	Cleaning and sanitising the work surfaces, setting out all the necessary equipment
The procedure	Following a set of instructions Adapting to different patients Adapting to available equipment Working in a systematic, clean way	Following recipes Adapting to varying produce Adapting to available equipment Working in a systematic, clean way
Working conditions	Time pressure, to resolve problem as quickly as possible Multiple patients, multiple needs Safe work with sharp tools Safe work with potentially dangerous equipment	Time pressure, to deliver food on time Multiple customers, multiple orders Safe work with sharp tools Safe work with potentially dangerous equipment

**FIGURE 1:**
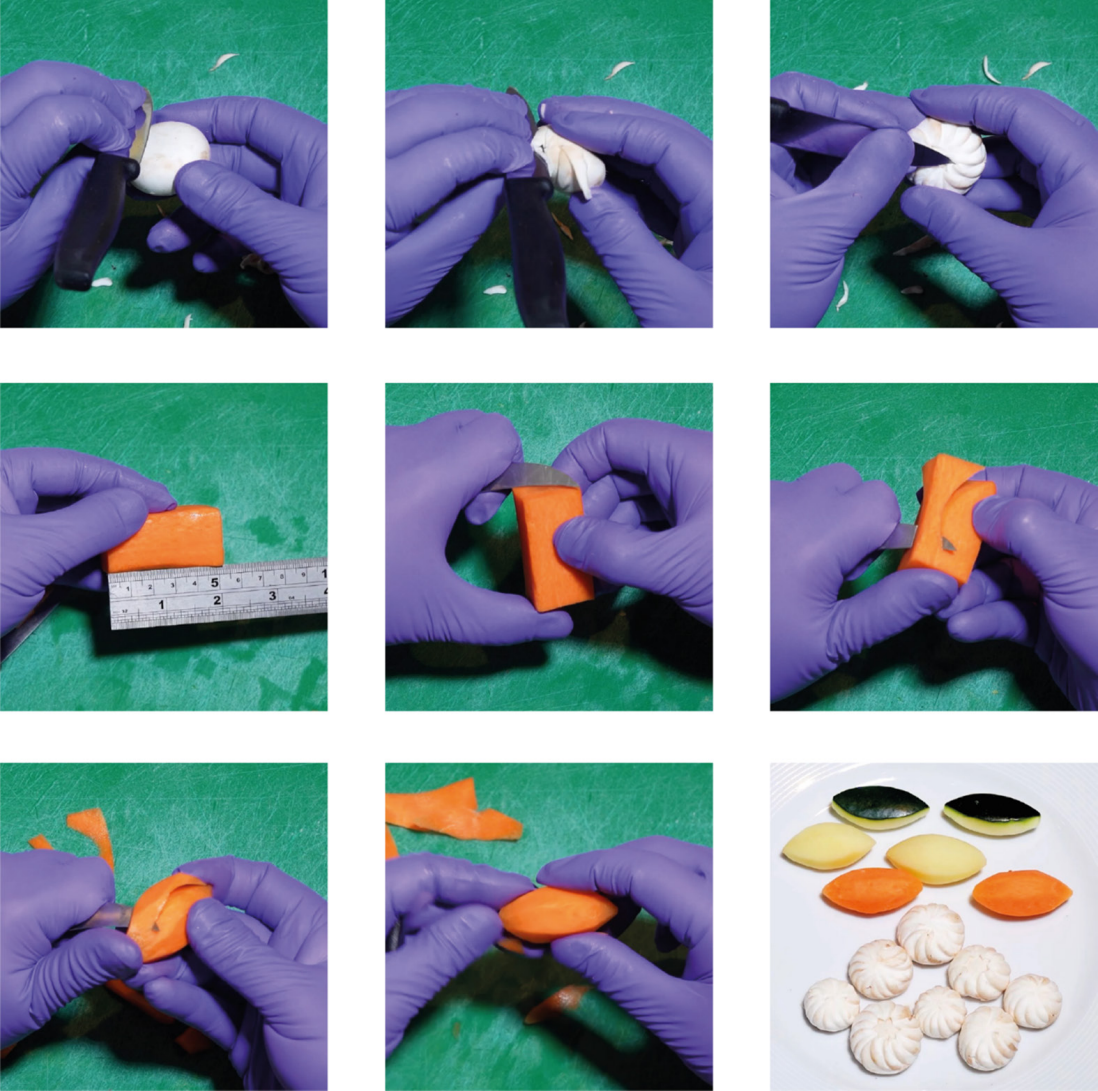
Turning mushroom and vegetables is a skill requiring precision, dexterity and observation. The top three pictures show the process of turning a mushroom to create a decorative effect, continued with the process of blocking off and turning a carrot to achieve a 7‐sided, 5 × 2 cm rugby ball shape. Bottom right—the final result of a practice run, with turned courgettes, potatoes, carrots and mushroom.

**FIGURE 2:**
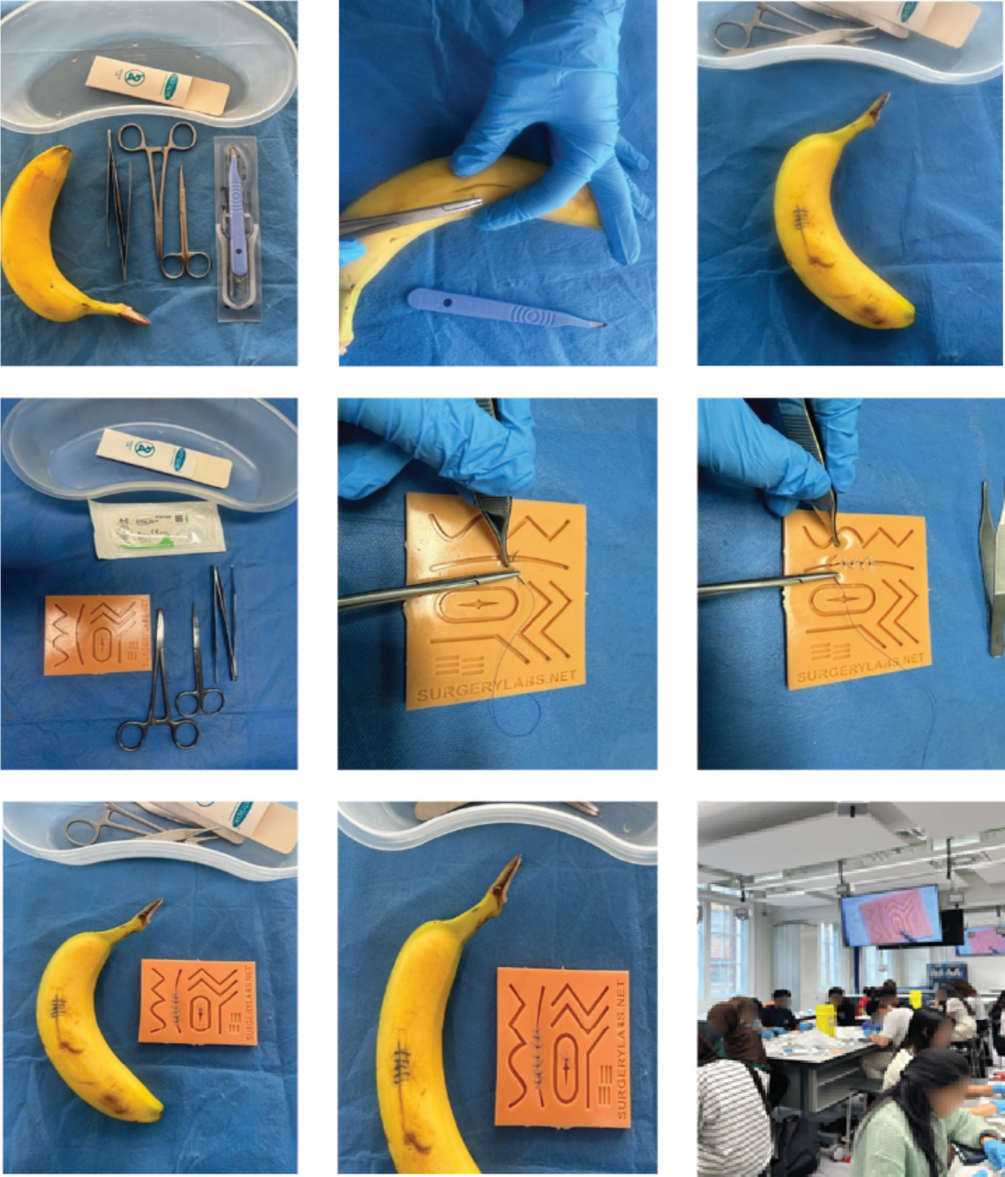
The surgical kitchen kit. The top row of images illustrates the practice of basic surgical knots using a banana, a method employed during the online sessions to simulate tissue handling. Progressing to the second and third rows, participants are seen advancing their technique by performing basic surgical knots on a silicone suture pad, emphasising the integration of effective communication skills throughout the process. The final image captures an in‐person session with medical students at Imperial College London, where these skills are applied in a collaborative learning environment.

We chose the skill of ‘turning vegetables’, using a knife to shape vegetables into consistent shapes (Figure 1)—which has parallels with suturing (Figure 2). This requires dexterity and control, gained through repeated deliberate practice, and presents similarities with clinical skills.

## 3 Evaluation

Qualitative data was gathered during two self‐reflective exercises, which were an integral part of the course. Each student was asked to fill out an online form, was presented with a participant information sheet about the study, and was asked to voluntarily opt in for their responses to be used for research. The data was gathered over 2 years, with approximately 360 students in each cohort. We have received a total of 375 responses with consent and analysed these using reflexive thematic analysis [15]. The authors (CSC, JLR) coded 863 text excerpts into 44 descriptive concept codes, which were audited by NH. After discussion, the codes were grouped into potential themes, refined into three major themes, assessed and presented below. The study and its methodology have been approved through the Education Ethics Review Process at Imperial College London (EERP2021‐079).

### 3.1 The Medical Kitchen Provides a Safe Environment for Learning Skills

The data indicated that MK supported practical skills learning (Quote 1, Table 3) and that the environment created was safe from the practical point of view—enabling students to practice the basics (Quote 2, Table 3), with particular emphasis on setting up the space for the procedure in an organised way (Quote 3, Table 3). In addition, the environment created a safe space for students to maintain a good, calm mindset during practice (Quote 4, Table 3). This theme highlights that a transdisciplinary practical intervention can provide an appropriate setting for practising manual skills. This is particularly important for practical considerations, as it is a low‐cost, accessible and easily executable approach that can be performed in person or remotely.

The data indicated that MK supported practical skills learning and that the environment created was safe from the practical point of view.

**TABLE 3:** Quotes from free‐response reflective exercise supporting the evaluation.

Quote no.	Quote
1	‘I was able to understand the turning technique and begin to apply this technique. I got used to creating a smooth curve using the turning knife which is something I found difficult at the beginning.’
2	‘[I did well] The basics like preparing the workspace and making sure to ensure hygiene during the procedure. During the actual procedure, i correctly and safely used the knife to turn the vegetables.’
3	‘I prepared well at the start and kept a clean station. I used correct PPE that was available to me.’
4	‘I demonstrated good basic techniques of suturing and remained optimistic during the activity, despite it being challenging at times.’, ‘I feel I was able to apply the skills demonstrated in the video calmly and methodically’.
5	‘I found that practicing the skill on my own was very different from presenting the skill to my partner due to the added pressure and the need to verbalise my actions and thought process.’
6	‘To improve, I would need to practise the process of turning vegetables‐ to not only improve my cuts, but to also make the explaining of the procedure more seamless with the doing of the procedure. This skill is very important in clinical practice.’
7	‘My assessor said I introduced myself and set up the station very professionally. This is good as doing this correctly means that patient will be very relaxed as I start the procedure.’ ‘I was able to explain the procedure well and gain the informed consent … I was professional and polite.’
8	‘Adapting the technique to different textures. This meant adapting the safety measures to ensure safe shaping technique. Having mastered using the knives on the soft texture of the courgette, the challenge of doing the same task on the carrot was interesting.’
9	‘I think that I could perhaps try and take it a bit slower and consider my next steps more carefully so that I do not rush in too soon. I also think that I could focus more on the process and worry less about the product especially at the beginning!’
10	‘In my next attempt I would slow down my pace and focus more on precision ‐ with more practice hopefully my technique would improve.’
11	‘On my next attempt I would perfect the placement of my knife on the vegetable surface before making the cut. My partner and I talked about what the most important step in the process and both agreed that it was knife placement; it seemed to influence the rest of the cut as well as being most pivotal in deciding the end shape.’
12	‘We persevered and we adapted ‐ the first time I made the knot I ripped through the banana, so we had to find different ways (eg. starting further away) so I would not rip it again.’

### 3.2 Beyond Technique—Communication, Professionalism and Adaptation

While practising clinical skills, it is pivotal that students develop skills to perform simultaneous communication with the patients. Students appreciated that doing while explaining is more challenging (Quote 5, Table 3) and understood that they must practice mastering simultaneous communication (Quote 6, Table 3). Moreover, students reflected on the importance of professionalism while doing procedures and informing the ‘patient’ to obtain consent (Quote 7, Table 3) and noticed how they need to adapt their technique to different vegetable textures. This is an important aspect of clinical practice, as different patients require adaptation of techniques (Quote 8, Table 3).

This theme highlights that a transdisciplinary practical intervention can teach skills and aspects adjacent to the practical skills and bring students attention to these skills and aspects early in their education so students can focus on them throughout their studies. Furthermore, students had the opportunity to understand the level of experience required to carry out a procedure while simultaneously communicating with a patient or colleague.

A transdisciplinary practical intervention can teach skills and aspects adjacent to the practical skills (…) early in their (students') education.

### 3.3 Practice and Performance—Learning Practical Skills

The Medical Kitchen aimed to help students develop a transferable approach to sustained deliberate practice and learning new psychomotor skills. Students learned to prioritise both process and results, recognising the importance of slow, focused training (Quote 9, Table 3) and noticed that focus and precision are important and that practice is needed for improvement (Quote 10, Table 3). Students have reflected on the need for deliberate, thoughtful practice to achieve success (Quote 11, Table 3). Additionally, they acknowledged the importance of perseverance and adapting to challenges in mastering practical skills (Quote 12, Table 3). This theme underscores that a transdisciplinary practical intervention like the Medical Kitchen can teach students a transferable approach to learning new psychomotor skills applicable to their clinical training. Although students remained in the cognitive phase of learning, the intervention helped them realise what is needed to progress to the associative phase and beyond.

Medical Kitchen can teach students a transferable approach to learning new psychomotor skills applicable to their clinical training.

## 4 Implications

The Medical Kitchen intervention provides a blueprint for aiding undergraduates as they start their clinical skills training. The transdisciplinary nature of the intervention provides a novel perspective on skill acquisition, which, with the support of reflective discussion, can be translated into useful insights for students' future learning. Future research could explore the long‐term impact of transdisciplinary interventions on students' clinical skill development, patient interactions and overall confidence as they progress through their medical education. Currently, the intervention has been integrated into the medicine curriculum at our institution and is being implemented at a collaborating university. We are focused on refining the Medical Kitchen intervention, as well as designing and implementing other transdisciplinary interventions in other areas of higher education.

The Medical Kitchen intervention provides a blueprint for aiding undergraduates as they start their clinical skills training.

Our transdisciplinary approach implemented in the Medical Kitchen course offers a promising way of addressing the challenges associated with teaching procedural skills in medical education. Through a safe, simple, cheap and easily implementable environment, it introduces the students to the concepts of learning a practical skill and deliberate practice, thus equipping them with essential knowledge needed in their future learning. It also makes them aware of the level of mastery required from a medical professional and provides a transferable approach to learning needed for success in clinical practice. Medical educators may consider implementing this innovative approach to introducing students to procedural skill learning to prepare and enhance the development of young medical professionals.

## Supporting information


**Data S1.** Supporting information.

## Data Availability

Research data are not shared. The Participant Information Sheet did not include provision for data sharing.
