# Supplementary material for: Medical Kitchen: Transdisciplinary Clinical Skills Training
Source: Clin Teach. 2025 Mar 8;22(2):e70065. doi: 10.1111/tct.70065 (PMC11889704; doi:10.1111/tct.70065)
Supplement: Supplementary file 1 — Data S1. Supporting information. [file TCT-22-e70065-s001.pdf]

IMPERIAL

Medical Kitchen

Lab Coordinators:

Dr Jakub Radzikowski

Dr Sofia Chacon Gonzalez

Prof. Roger Kneebone

Dr Aynkaran Dharmarajah

Dr Preeshila Behary

Lab developers:

Dr Oliver Armstrong-Scott

Dr Natasha Houghton

Co-developed with Sarah Herler through the StudentShapers programme.

Aims of Medical Kitchen

Competence in practical procedures (like cannulation) is essential when you begin clinical attachments. But developing the necessary confidence and dexterity to handle unfamiliar equipment and do practical tasks with your hands is challenging. An additional layer of complexity is added as you must communicate appropriately with patients in real time during procedures. This can be daunting, causes anxiety, and even makes some people avoid doing practical procedures.

The world of gastronomy uses much of the same expertise. Chefs, when working in the kitchen, set up their workplace with the necessary tools. They work with potentially hazardous equipment while keeping things clean and safe, often under intense time pressure. They follow procedures which require a high level of craftsmanship and manual dexterity to produce a consistent outcome. And they communicate constantly with others throughout the process to ensure the smooth running of the kitchen.

The "Medical Kitchen" is a course where you will put these skills into practice using cooking and the principles of gastronomy at the university. Isolating these key skills from the high-stakes medical environment lets you improve in a context where mistakes do not need to cause anxiety and where being less than perfect won't feel like a disaster. We hope you have some fun along the way!

Click here to see a short video introducing the programme.

Scan with a phone camera or click and watch the video!

Learning objectives

1. Interpret and follow protocols for an unfamiliar procedure with an appropriate level of planning and forward thinking before beginning practical work
2. Handle equipment – safely prepare, use and clean potentially hazardous equipment
3. Demonstrate safe and hygienic working practices using aseptic technique where appropriate
4. Practice meticulous technique with a high level of dexterity, working carefully to manipulate tools consistently
5. Communicate appropriately while completing the procedure describing it in real time
6. Document outcomes accurately, in appropriate details and in an organized manner
7. Reflect on constructive feedback from self-reflection, peers, and tutors – learn from errors, seeing them as part of the process of skills development

Programme Overview:

This course is composed of 2 in-person sessions.

Session : The Medical Kitchen

You will learn to turn vegetables; you will practise this skill and then perform it for one of your peers who will grade you on your performance. You will have chance to discuss your experience during a short reflective discussion.

Practising a New Skill

Format: independent learning

Duration: 15 minutes

Description: During this activity, you will watch instructional videos on how to perform the new skill. You will then practise doing this yourself until you are confident that you are ready to perform it to one of your peers.

Performing a New Skill

Format: learning in pairs

Duration: 15 minutes (20 minutes per person)

Description: you will need to perform your new skill in real time with the additional pressure of someone watching (and marking you). You will need to communicate appropriately while performing the procedure, pretending that your peer is your patient / customer. You will be provided with a marking sheet to assess your peer. All marks are formative and do not count towards your final grade. At the end of this activity, you will complete a reflective exercise.

Short reflective discussion

Duration: 15 minutes

During this discussion, we will answer any immediate questions and explore how skills you learned during independent learning can be applied in practice and consider why this is important, in preparation for Session 3.

Session 2: Application of Session 1 learning to suturing

You will learn basic concepts of suturing techniques; you will practise suturing on a silicone suture pad and then perform it for one of your peers (communicating with them as though they were the patient). They will formatively grade you on your performance.

Practising a New Skill

Format: independent learning

Duration: 30 minutes

Description: During this self-directed activity, you will watch instructional videos on how to perform the new skill. You will then practise doing this yourself until you are confident that you are ready to perform it to one of your peers.

Performing a New Skill and debriefing

Format: learning in pairs

Duration: 30 minutes

Description: you will need to perform your new skill in real time with the additional pressure of someone watching (and marking you). You will need to communicate appropriately while performing the procedure, pretending that your peer is your patient / customer. You will be provided with a marking sheet to assess your peer. All marks are formative and do not count towards your final grade. At the end of this activity, you will complete a reflective exercise.

Reflective discussion - debriefing

During the debriefing, we will explore how skills you learned during independent learning can be applied in practice and consider why this is important. We will also be answering your questions - you can ask them yourselves, or submit anonymously through a mentimeter link displayed on the day.

Session 1: The Medical Kitchen

During part 1 you will be introduced to a couple of classic decorative knife skills. You will complete a set of tasks to create a set of classic French decorative cuts – from a variety of vegetables. You should practice this skill until you are comfortable with the technique. Click on the QR code on the right to watch a short video exploring the similarities between classic knife skills and clinical procedures.

Scan with a phone camera or click and watch the video!

Kitchen safety and food hygiene

The main risk associated with professional cooking is the cross contamination of food with bacteria, viruses or fungal pathogens. Some foods, like raw meat, soil-covered vegetables and others, can be covered in these pathogens that can cause, among others, gastrointestinal disorders. Proper preparation, usage of colour coded equipment for different classes of products, cleanliness of the workplace and proper food storage are thus very important in ensuring food safety.

Here are videos prepared by the Food Standards Agency (an UK body regulating Food Safety Standards) explaining how cross-contamination can happen during food preparation:

Surprisingly though, the main source of contamination in the kitchens is the chef. This is why personal hygiene, proper procedure and avoiding "bad habits" such as licking fingers and touching your face – hygienic working practice – as well as PPE and proper attire are the most critical elements in making sure the food prepared is safe to eat.

Scan with a phone camera or click and watch the video!

Scan with a phone camera or click and watch the video!

Proper attire

For personal safety and food safety, a chef should:

1. Wear an apron, long trousers that cover ankles, full shoes with full length socks, and preferably a double-breasted jacket when working with hot products.
2. Not wear earrings, as they might fall into the food; and keep hair tied or under a hat/hairnet to avoid getting it entangled.
3. Not wear bracelets, watches, rings, have long or varnished fingernails, as these are potential reservoirs of pathogens.

Preparing for cooking

Washing your hands

In a kitchen, wearing gloves is not essential – it is mostly done for comfort and protecting yourself from, for example, food that can stain your hands such as beetroots or artichokes, and not to protect the food from yourself. Properly washed hands are a safe tool to use in the kitchen, and a proper washing technique must be employed.

Please refer to the video linked on the right and prepared by the Food Standards Agency (an UK body regulating Food Safety Standards) to learn the proper technique.

Scan with a phone camera or click and watch the video!

Cleaning your workstation

The work surfaces that you will use for cooking need to be cleaned and sanitized – they can be covered with pathogenic bacteria that can contaminate the food. Please refer to the video linked on the right to see the proper cleaning technique:

Scan with a phone camera or click and watch the video!

Setting up your workstation

In order to work in an efficient, clean way, you need to set up your workstation properly. You will need your tools, your ingredients, and receptacles for waste, trimmings and ready made products. Such preparation is called mise-en-place in the culinary world. We have prepared a video explaining this concept, using the procedure of chopping an onion as an example:

Scan with a phone camera or click and watch the video!

Kitchen safety rules

1. If you have any food allergies, you must make sure you do not use these ingredients in your experiments. If you have any questions or concerns about that, please contact the workshop coordinators.
2. Knives are sharp. Do not cut yourself. In case you do, stop working, inform your tutors, and make sure you get appropriate medical attention if needed.
3. Even though you are working in your own kitchen, you must be dressed correctly.
4. Wear flat-bottomed, closed toe shoes. No sandals, flipflops or high heels.
5. Wear long trousers or a skirt that covers the ankles. No shorts or short skirts.
6. Wear a top with sleeves like a t-shirt or longer. No vests, spaghetti straps etc.
7. If you have long hair, it must be tied at the back.
8. Do not chew gum or smoke in the kitchen area.
9. Do not run in the kitchen area.
10. Do not wear headphones or music players in the kitchen area. You must be aware of your surroundings to work safely.
11. You must wash your hands with soap upon entering the kitchen area. You must also wash your hands after sneezing, coughing, licking your fingers, touching your nose etc.
12. Clean as you go. Wash your tools and bench space. Keep everything tidy and organized. Dispose of waste properly using the general waste, food waste or recycling containers as appropriate.
13. Do not "double-dip". If you use a spoon to taste something, wash the spoon before tasting again.
14. Store all food in the fridge.
15. If you have any questions concerning safety or any other aspect of the activities - please contact the course coordinators. We are happy to help you and answer your questions.

Activity One: Practising a New Skill

This activity can be completed independently. You will be introduced to "basic knife skills". Like many clinical procedures, these skills look simple. It's easy to understand what you are meant to do but quite difficult to actually do it!

Background:

Your first task is to learn to how to turn a carrot, potato or a courgette (or, if these are unavailable, a similar root or tuber vegetable).

Recommended Technique:

1. Prepare yourself and your workspace as outlined in the Kitchen safety and food hygiene section of this manual. In particular:
   a. Wash your hands
   b. Remove any earrings, bracelets, rings, etc. and tie your hair
   c. Clean your workspace
   d. Wash your hands
   e. Set up your workstation
   f. Wash the vegetables
   g. Wash your hands
2. Make sure you have all your equipment and ingredients prepared and that you have read through this procedure completely before attempting to practise the techniques.
3. Turn the potatoes. Then switch to courgettes. The technique is explained in the following video (QR code at the bottom of the page).
4. The turned vegetables (potatoes or courgettes) should be about 5cm long, 2cm in diameter and be in the shape of a 7-sided rugby ball. Use a ruler to check the dimensions.
5. Separate your trimmings from your ready-made products using the provided receptacles.
6. After learning the first skill, clean your tools and workstation. Make sure you dispose of all vegetables in the food waste bin. Do not put anything non-biodegradable in the food waste bin.
7. Document your progress, from the first turned vegetable to the last – take pictures and compare them.

Scan with a phone camera or click and watch the video!

Activity Two: Performing Skills Under Pressure

After completing the first activity, you should be familiar with the basic knife skills techniques and be able to create a turned potato/carrot.

But doing this with someone assessing you, when you must focus on communication as well the steps and when you have a strict time limit, is far harder than practising in complete privacy!

You should:

1. Demonstrate the skills you learned in activity one for your partner, while simultaneously talking through the process in a step by step fashion. You should imagine that you are teaching your partner how to do the procedure and describe each step as you go.
2. While you are doing this, your partner will complete the skills marking sheet (appendix 1)
3. Swap roles and repeat the process
4. Reflect on your performance and receive feedback from your partner.
5. Appropriately document the procedure and complete the guided reflection
6. Submit your guided written reflection to the facilitator.

Session 2: Suturing

In part one you learned a new practical culinary task. We talked about the importance of preparing and how it is essential to make your environment and equipment work for you. We explored how to handle potentially hazardous equipment, and how to clean up safely when you finish. We looked at how repeated practice is needed, when something looks simple, but is much harder than it looks. And we asked to perform your new skills for one of your peers, to practise communicating at the same time.

Now we take all these key concepts and apply them to a core clinical procedure, suturing.

But before we get started, we need to talk about using your equipment safely and setting up your sterile field.

Safety when suturing

Like working in the kitchen, the main risk during a simple procedure (such as suturing) is cross contamination which can cause infection. To minimise this risk, you need to use sterile equipment, set up a "sterile field" before starting the procedure, and use aseptic technique during the entire procedure. This is discussed below.

The other risk is minor injuries from equipment (needle stick injury). This is particularly dangerous if the needle is "contaminated" (i.e., has been in contact with the patient's blood or tissue). In this case a needle stick injury would result in a blood and body fluid exposure. BBF exposures need to be thoroughly followed up to ensure that there has been no transmission of bloodborne viruses. Each hospital or clinical site will have a protocol for managing BBF exposures. As you will not be suturing humans, it is very unlikely that you will have a BBF exposure during this course. However, it is still essential that you properly dispose of your equipment to prevent you or others injuring themselves with unclean needles.

If for any reason you do have a BBF exposure* during these activities, please contact the co-ordinator for this course immediately by emailing:

Chacon Gonzalez, Sofia ‹c.chacon-gonzalez@imperial.ac.uk›

Radzikowski, Jakub L ‹j.radzikowski@imperial.ac.uk

If you have latex allergy, please let the tutors know prior to commencing this activity.

*An example of this might be if you accidentally stabbed yourself with a needle and then left it sitting on the bench and your house mate or family member subsequently stabbed themselves with the same needle.

Disposing of Sharps

Please do not let anyone else "practise" with your needles. They should only be handled by you.

It is essential that you dispose of your suture needle as soon as you have finished the section in the sharps bin.

Aseptic Technique and the Sterile Field

What is aseptic technique?

Aseptic means "without microorganisms", in other words completely clean and without contamination. Aseptic technique is the way we perform clinical procedures without contaminating our patients (or ourselves) in any way. Important aspects of aseptic technique include:

1. Through handwashing (or scrubbing for surgery)

2. Creating a "sterile field" by laying out sterile drapes (fresh out of the packet), placing all of your equipment into the sterile field (without contaminating it)

3. Cleaning your patients' skin (with chlorhexidine or similar) and then setting up a sterile field over their skin so that only the area you need to access is visible

4. Wearing suitable PPE equipment (sterile gloves for suturing). You should also be "bare below the elbows". This mean not wearing any clothing below the elbows, rings, or nail polish as these can carry pathogens.

5. Not contaminating either your gloves or your sterile field during the procedure

This is harder than it sounds, you are not sterile (even if you wash your hands), so you cannot touch your sterile field unless you are wearing sterile gloves (even if you are setting it up). Once you have your gloves on you cannot touch anything other than your own sterile field! This is easiest to understand when you watch how it is done.

Activity One: Practising a New Skill

This activity can be completed independently in your own kitchen. You will be learning to do the most basic type of suturing known as a simple interrupted suture.

Background:

Your first task is to learn to suture up a 5cm wound. Today your patient will be a suture pad! Just as with part one, you should practise this skill until you are confident that you can do it well enough to perform in front of your partner.

Equipment:

You have been sent an equipment pack which includes:

1. Sterile suture kit: this contains 1 pair of skin forceps, 1 needle holder, 1 pair of scissors, 1 sterile drape, 5 gauze
2. Gloves (these are not sterile gloves; with a real patient you would use sterile gloves)
3. Suture (with needle)

Please set up your sterile field, try to use aseptic technique as shown in the video above. Once your field is prepared, place the suture pad in the sterile field (you can pretend it is sterile and assume it has been thoroughly cleaned).

Today we will only be asking you to suture. However, in real life it is important that you use local anaesthetic to anesthetise the skin before suturing. The demonstration video includes instructions on how to do this, but you do not actually need to anaesthetise your patient today because it is a piece of silicone.

Recommended Technique:

The correct technique can be viewed in this video:

Before and after each patient contact or procedure clean hands as appropriate with soap and water or alcohol gel.

1. Identify the patient.
2. Discuss the procedure with the patient and obtain informed consent.
3. Clean your hands.
4. Clean your workspace and collect your equipment.
5. Open dressing pack and empty out other required equipment on to the sterile field.
6. Wash your hands, including wrists and distal third of forearms.
7. Apply gloves (once you are wearing gloves you are “sterile” for this procedure, with an actual patient these need to be sterile gloves).
8. Organise layout of items on sterile field.
9. Map out where you plan to insert your sutures, noting the position of wound edges and skin folds.
10. Insert the first suture in the middle of the wound and then continue dividing into equal sections – approximately 5-10mm apart.
11. Grasp the needle two thirds of the way from the needle’s point with the needle holder.
12. Holding the suture pad with toothed forceps, pierce the skin at a 90° angle not closer than 5mm from the wound edge, following the curvature line of the needle as it passes through the tissue, into the middle of the wound - (you should aim to go through the whole length of the suture pad without actually damaging the suture pad itself).
13. Remove the needle and remount it in the needle holder before taking a corresponding bite on the other side of the wound. Do not touch with fingers to avoid needle stick injury. Hold the needle with toothed forceps while repositioning it in the needle holder.

Scan with a phone camera or click and watch the video!

14. Pull the suture through until approximately 15cm remains.
15. Tie a surgical knot – ensuring that all knots end up on the same side.
16. Cut both ends of the suture to an appropriate length e.g., 5-10mm.

17. Clean the wound and apply gentle pressure to ensure bleeding ceases.
18. Take note of the number of sutures before applying a non-adherent dressing.
19. Instruct the patient appropriately, including precautions and aftercare.
20. Dispose of equipment safely and appropriately and remove gloves.
21. Clean hands.
22. Document the procedure in the patient notes including, suture material, number of sutures and follow up information given.

Please note this procedure has been adapted from the CMS guide to practical procedures. It has had some steps removed to make it suitable for easy practice. For the full technique, as it should be performed on a patient, please refer to the CMS guide to practical procedures.

Activity Two: Performing Skills Under Pressure

After completing the first activity, you should be familiar with how to close a wound using simple interrupted sutures.

But doing this with someone assessing you, when you must focus on communication as well as the steps and when you have a strict time limit, is far harder than practising in complete privacy!

In this section you will work together in pairs in the MDL lab. You should:

1. Demonstrate the skills you learned in activity one for your partner, though you will still be suturing a suture pad, you should pretend that your partner is the patient. Please make sure you introduce yourself, explain the procedure and get informed consent!
2. While you are doing this, your partner will complete the skills marking sheet (appendix 1)
3. Swap roles and repeat the process
4. Reflect on your performance and receive feedback from your partner using the debriefing template (appendix 1)
5. Appropriately document the procedure and complete the guided reflection
6. Submit your guided written reflection to the facilitator

This activity should take approximately 1 hour.

Activity Six: Debriefing

You must submit your guided reflection and documentation of their procedure prior to the whole group wrap-up session. Discussion will focus on how learnings can be applied in medicine.

You can also submit question prior to the debrief - we will provide you with a mentimeter link to submit them anonymously.

Appendix One

Marking Sheet - decorative vegetable cuts

1	*Introduction*	
	1. Checks partners name and states role and purpose (cook demonstrating turning skills)<br>2. Washes hands	
2	*Workspace preparation*	
	3. Selects correct equipment and food required for the task<br>4. Sets up before beginning<br>5. Does not need to stop during the procedure to collect extra equipment	
3	*Food Safety and Hygiene*	
	6. Correct procedure during set up – including washing vegetable, workspace and hands<br>7. Hygiene and food safety maintained throughout procedure<br>8. Appropriate infection control and use of PPE	
4	*Turning Procedure*	
	9. Handles equipment appropriately throughout<br>10. Follows turning steps in order<br>11. Completes the process having successfully turned the vegetable	
5	*Communication*	
	12. Professional and polite<br>13. Provides a consistent and accurate narrative of each step during the process in real time (ideally you should be able to follow what they are doing even if you aren't looking).	
6	*Closes*	
	14. Safely disposes of equipment and washes hands<br>15. Offers to answer any questions about the process<br>16. Describes information that they would record	

Marking Sheet - suturing

1	*Introduction*	
	1. Checks partners name and states role and purpose<br>2. Explains procedure<br>3. Gain informed consent<br>4. Washes hands	
2	*Workspace preparation*	
	5. Selects correct equipment required for suturing<br>6. Sets up before beginning<br>7. Does not need to stop during the procedure to collect extra equipment	
3	*Aseptic Technique and Safety*	
	8. Correct procedure during set up – sets up without contaminating field<br>9. Aseptic technique maintained during procedure<br>10. Appropriate infection control and use of PPE<br>11. Safely disposes of sharps	
4	*Procedure and Technique*	
	12. Handles equipment appropriately throughout<br>13. Follows suturing steps in order<br>14. Completes the process having successfully closed the wound	
5	*Communication*	
	15. Professional and polite<br>16. Provides a consistent and accurate narrative of each step during the process in real time (ideally you should be able to follow what they are doing even if you aren't looking).	
6	*Closes*	
	17. Safely disposes of equipment and washes hands<br>18. Offers to answer any questions about the process<br>19. Describes information that they would record	
